# Exploring the Association Between Physical Activity, Regulatory Emotional Self-Efficacy, Perceived Self-Burden, and Social Isolation Among Older Adults in China

**DOI:** 10.3390/healthcare13060687

**Published:** 2025-03-20

**Authors:** Shicheng Yang, Huimin Peng, Longjun Jing, Huilin Wang, Shuyin Chen

**Affiliations:** 1 School of Physical Education, Hunan University of Science and Technology, Xiangtan 411201, China; 2 School of Business, Hunan University of Science and Technology, Xiangtan 411201, China

**Keywords:** physical activity, regulatory emotional self-efficacy, self-perceived burden, social isolation, older adults

## Abstract

**Background**: As people age, their physical functions decline, and changes in social roles and life experiences put older adults at a higher risk of social isolation. **Methods**: In this study, we employed both snowball and purposive sampling techniques to collect valid data from 237 seniors aged 60 to 75 residing in Hunan Province. Using structural equation modeling (SEM) with the partial least squares (PLS) method, we examined the relationship between physical activity and social isolation among this elderly population. **Result**: Findings from this study indicate a positive association between physical activity and regulatory emotional self-efficacy, while revealing a negative association with the perception of being a burden to others. Moreover, a higher regulatory emotional self-efficacy is linked to reduced social isolation, whereas an increased sense of self-perceived burden is associated with greater social isolation. This study also uncovers that the link between physical activity and reduced social isolation is influenced by both regulatory emotional self-efficacy and the perception of self-burden. **Conclusion**: It is imperative for a collaborative effort involving government entities, societal groups, family units, and individuals to meticulously address and cater to the diverse requirements of senior citizens.

## 1. Introduction

The growing aging population has highlighted the issue of social isolation among the elderly, driven by societal shifts, changes in family dynamics, and the seniors’ own physical and mental factors. Numerous studies have shown that health problems are a major challenge currently faced by older adults, such as declining physical functions and increasing diseases, which not only affect their quality of life but also easily lead to psychological panic [1]. According to statistics, about 80% of older adults in China suffer from one or more chronic diseases [2], which not only affect their physical health but also bring many inconveniences to their lives. Moreover, the psychological well-being of elderly individuals is increasingly compromised by feelings of loneliness, loss, and helplessness, which can escalate into emotional conditions like depression and anxiety [3]. Secondly, the social problems of older adults are also severe. The departure of their children and the loss of professional networks post-retirement make it challenging for elderly individuals to form new social connections. This situation often results in heightened experiences of loneliness and feelings of being sidelined [4]. In addition, the relatively low level of pensions often puts them under great pressure in terms of living [5]. A combination of these issues is a significant factor in causing social isolation among older adults, and if not addressed promptly and effectively, the problem will become increasingly significant.

Appropriate physical activity has many benefits for older adults, including enhancing cardiopulmonary function, preventing or delaying the onset and progression of chronic diseases [6], and alleviating symptoms of depression [7]. Additionally, engaging in sports not only enhances the psychological fortitude of elderly individuals, assisting them in managing their emotions effectively, as highlighted by Fox [8], but also offers them chances to meet and befriend new people, thereby widening their social networks, according to Dionigi [9]. Australia has introduced policies to promote physical activity among older adults, providing a supportive physical environment for them [10]. The publication of policy frameworks like the “National Fitness Plan (2021–2025)” underscores China’s emphasis on promoting physical activity among its aging population. To adapt to new trends and fully leverage the positive role of sports in addressing population aging, China will further strengthen the guidance and coordination of physical activities for older adults, ultimately alleviating social isolation among them at its root. Global and national guidelines recommend that older adults should ideally partake in at least 150 min of moderate-intensity physical activity per week. However, diverse factors including personal preferences, cultural norms, and historical contexts can lead to significant differences in the types, approaches, and venues of physical activities practiced by older individuals [11]. Therefore, older adults can choose to participate in different levels and types of sports activities according to their actual situation, ranging from leisure sports activities such as walking and jogging, to higher-level intense exercises like master-level running and swimming [12], all of which can bring many benefits to older adults.

Despite the implementation of various policies by the government to assist the physical activities of older adults, the effects are not yet significant. The reasons for this phenomenon are multifaceted, such as discrepancies in policy formulation and execution, lack of effective monitoring and evaluation mechanisms, insufficient resource investment, low social participation, and factors related to older adults themselves. Therefore, providing consistent and prompt updates on the real-world conditions and challenges to governmental bodies and pertinent agencies is crucial for enhancing the efficacy of policy enactment. In their research, scholars posit that the Chinese government has developed a broad set of policies and urge the elderly to acknowledge the significance of engaging in physical activities. Additionally, the reasons for social isolation among older adults are not limited to the social level but are more importantly linked to individual psychological states [13]. Studies have shown that when entering old age, older adults face multiple factors such as changes in living environment, loss of relatives and friends, and health troubles, which can lead to increased psychological stress and burden, thereby affecting their social behavior and interpersonal relationships [14].

For older adults, maintaining an optimistic attitude is crucial, as it can better promote both physical and mental health, ultimately enhancing overall quality of life [15,16]. Particularly during health crises, the mental well-being of elderly individuals can undergo dramatic shifts. Chronic conditions may not only deplete their physical strength but also provoke adverse emotional responses like loneliness, anxiety, and depression, as observed by Girdhar, Srivastava, and Sethi [3]. These accumulated negative feelings, coupled with challenges in expressing them, can detrimentally impact seniors’ physical and mental health. Furthermore, such circumstances can intensify obstacles in communicating with family and friends, further isolating them in their social interactions. Currently, research on the social isolation of older adults has attracted widespread attention. However, existing studies mostly focus on the concept, measurement methods, influencing factors, and psychological and physiological consequences of social isolation [17,18], and propose various intervention measures for the issue [19]. Unlike previous studies, this study focuses for the first time on how physical activity can reduce the degree of social isolation by enhancing regulatory emotional self-efficacy and alleviating self-perceived burden. This research bridges the connection between psychological aspects and physical activities, addressing the voids left by prior studies, and quantitatively elucidates how regulatory emotional self-efficacy, the sense of self-perceived burden, and social isolation are interrelated. Consequently, the goals of this investigation are multifaceted: (1) to examine how physical activity influences social isolation in elderly populations; (2) to investigate the interplay between physical activity, the ability to manage emotions, perceived personal burden, and the experience of social isolation; and (3) to offer recommendations for mitigating the issue of social isolation among the elderly.

This research centers on the social disconnection experienced by elderly individuals, delving into the effects of physical well-being on mental health. It advocates for engagement in physical exercises as a strategic approach to alleviating social isolation within this demographic. The specific path analysis is as follows: from simple leisure sports activities, such as walking and jogging, to higher-level intense exercises, such as master-level running and swimming, all of which can help older adults regulate emotions, alleviate psychological burdens, and promote social communication. This research underscores that regulatory emotional self-efficacy and the perception of self-perceived burden act as mediators in linking physical activity with social isolation. This suggests that elderly individuals with higher capabilities in managing their emotions experience less social isolation. Similarly, those who perceive themselves as less of a burden exhibit greater emotional self-regulation skills and, consequently, face reduced social isolation. Thus, grasping how physical activity affects the social disconnection experienced by the elderly not only enriches theoretical understanding but also prompts increased focus from governmental bodies, societal institutions, and family units on the physical and psychological well-being of older adults. It further advocates for widespread participation in physical activities.

## 2. Literature Review and Hypothesis Development

### 2.1. Concept of Variables

#### 2.1.1. Physical Activity

Physical activity refers to direct participation in sports or indirectly engaging in a series of activities related to sports, including both direct and indirect involvement [20]. The European Commission categorizes sports as engaging in any physical activities in a leisurely or organized manner with the goals of enhancing or showcasing physical capability and mental health, forging social ties, or achieving competitive outcomes at various levels, which may involve solo or team efforts [21]. Similarly, physical activity serves as an essential tool for managing life’s stresses and sustaining mental health, offering benefits like stress reduction, mood regulation, improved social connections, and is deemed vital for fostering mental health progress [22].

#### 2.1.2. Regulatory Emotional Self-Efficacy

Caprara et al. [23] define regulatory emotional self-efficacy as the belief in one’s own capacity to successfully manage and control their emotional responses. It is a form of thinking centered on oneself, a belief, judgment, or subjective self-perception that an individual has before performing a certain behavior or action about their capability to complete that action at a certain level [24]. This research examines the concept of regulatory emotional self-efficacy, which is understood as the self-perceived capacity for managing and articulating both positive and negative emotions.

#### 2.1.3. Self-Perceived Burden

Bigger and Vo [25] describe self-perceived burden as a state in which individuals feel guilt and anxiety over the financial and emotional strain their illness might place on others and their families. This concept, initially formulated by Cousineau et al. [26], is seen as a complex notion stemming from the dependency of those receiving care and the resultant stress and concerns, potentially leading to feelings of guilt towards those providing care. Smith et al. [27] linked self-perceived burden to the adverse emotions experienced by people suffering from various conditions, highlighting its impact not just on the patients’ quality of life but also on their families and wider society, as supported by Yeung et al. [28].

#### 2.1.4. Social Isolation

Social isolation, the condition in which a person seldom or never engages with wider society, leads to detachment from societal and interpersonal connections. This condition is typically seen as detrimental to psychological health, linked to loneliness, depression, anxiety, and various emotional challenges [29]. The concept posits that social isolation can adversely influence one’s mental well-being, heighten the likelihood of heart and brain diseases, and reduce social skills [30]. It is also crucial to recognize that perceptions of social isolation can vary significantly among individuals, highlighting its subjective nature [31].

### 2.2. Hypothesis

Existing research suggests that individuals possessing strong regulatory emotional self-efficacy are adept at adapting their emotional regulation tactics in response to shifts in their surroundings, allowing them to promptly adjust and sustain a positive emotional condition [32]. When older adults are confident in their emotional management abilities, they are more willing to participate in social activities, establish connections, and interact with others [33]. In their analysis, Wang et al. [34] investigated regulatory emotional self-efficacy as a bridge linking physical activity and stress perception. The findings indicate that regulatory emotional self-efficacy plays a crucial intermediary role, with a discovered inverse relationship between physical activity and the level of stress perceived. In addition, studies including researcher Nes et al. [35] has connected self-perceived burden with factors like life quality, health status, and treatment approaches, alongside a notable correlation with physical activity. Brown and Gilmore [36] emphasized the significance of lifestyle and physical activity for cancer survivors, indicating improvements in survival rates, quality of life, and a decrease in disease recurrence risks and self-perceived burden. These findings propose that physical activity could serve as an effective strategy to mitigate self-perceived burden, though the specific interventions and outcomes may differ based on individual and environmental factors. In summary, the following hypotheses are proposed for this study:

**Hypothesis** **1** **(H1):** *Physical activity is positively correlated with regulatory emotional self-efficacy*.

**Hypothesis** **2** **(H2):** *Physical activity is negatively correlated with self-perceived burden*.

Previous studies have shown that the ability to effectively manage emotions plays a crucial role in personal mental well-being and the ability to handle stress and anxiety [37]. Individuals who possess a strong capacity for emotional regulation are more adept at navigating through stress and adversities, often maintaining a more optimistic outlook and emotional balance, which in turn diminishes the likelihood of experiencing psychological issues [38]. People with high self-perceived burden may feel more tired, helpless, and depressed, and this also affects their ability to cope with stress and challenges [39]. This suggests a link between the ability to manage emotions effectively and the perception of oneself as a burden. Berking et al. [40] found that interventions in emotional regulation can help individuals improve regulatory emotional self-efficacy, thereby improving personal mental health status and quality of life. These interventions may include emotional regulation training, cognitive restructuring, and psychological education. Specifically, when individuals are confident in their emotional management abilities, they may feel less stress and burden in life [41]. Conversely, if individuals lack regulatory emotional self-efficacy, they may feel more stress and burden and find it difficult to cope with these challenges [42]. In summary, the following hypotheses are proposed for this study:

**Hypothesis** **3** **(H3):** *Regulatory emotional self-efficacy is negatively correlated with self-perceived burden*.

According to Bandura’s theory, self-efficacy plays a crucial role in determining health outcomes, acting as a mediator [43]. Evidence indicates that a robust sense of self-efficacy serves as a protective mechanism, mitigating the risks of depression and social isolation. Individuals with stronger confidence in their self-efficacy tend to report lower levels of loneliness and depression [44]. Moreover, self-efficacy is pivotal for maintaining a healthy lifestyle [45]. The ability of elderly individuals to regulate their emotions, or their regulatory emotional self-efficacy, has a direct impact on their social isolation levels [46], indicating a link between regulatory emotional self-efficacy and social isolation.

As they age, the social circles of older adults tend to shrink. Influenced by changes in family relationships, declining physical function, neglect of psychological needs, and insufficient social support, they are prone to social isolation. Research indicates that chronic disease patients with a heightened sense of self-perceived burden tend to experience greater social isolation, suggesting a possibly two-way relationship where social isolation can intensify the perceived burden of disease and its treatment, which in turn may worsen social isolation [47]. Additionally, Hill and Frost [48] explored how self-perceived burden, the search for social support, and the diversity of social networks affect loneliness and psychological distress in women with ovarian cancer. They concluded that self-perceived burden and social network diversity are key factors contributing to the loneliness and psychological distress experienced by these women, significantly impacting their mental well-being. These studies collectively highlight the intricate link between self-perceived burden and social isolation, suggesting that alleviating perceived burdens could help diminish social isolation, thereby enhancing social interaction capabilities and quality of life. Therefore, based on past research, this paper proposes the following hypothesis:

**Hypothesis** **4** **(H4):** *Regulatory emotional self-efficacy is negatively correlated with social isolation*.

**Hypothesis** **5** **(H5):** *Self-perceived burden is positively correlated with social isolation*.

Prior studies have identified a link between physical activity and social isolation [49]. On one hand, physical activity can alleviate social isolation, promote individual interactions and connections with others [50], provide opportunities to meet new friends, increase social circles, and reduce feelings of loneliness [51]. Conversely, social isolation might lead to decreased physical activity as isolated individuals could find themselves lacking the drive and self-assurance necessary for engaging in physical or sports activities, resulting in lower levels of such activity [52]. Previous inquiries have established a bidirectional influence between physical activity and social isolation without delving into the potential role of intermediary factors. Integrating findings from various studies suggests that physical activity beneficially influences emotional self-regulation and lessens the sense of self-perceived burden. This perceived burden has a direct impact on social isolation levels. Additionally, social isolation can adversely affect mental health, whereas enhancing emotional self-regulation capabilities can diminish feelings of isolation. This raises the question: could emotional self-regulation and perceived burden act as mediators in the relationship between physical activity and social isolation? Based on this, the study proposes the following hypothesis:

**Hypothesis** **6** **(H6):** *Regulatory emotional self-efficacy and self-perceived burden mediate the relationship between physical activity and social isolation*.

**Figure 1:**
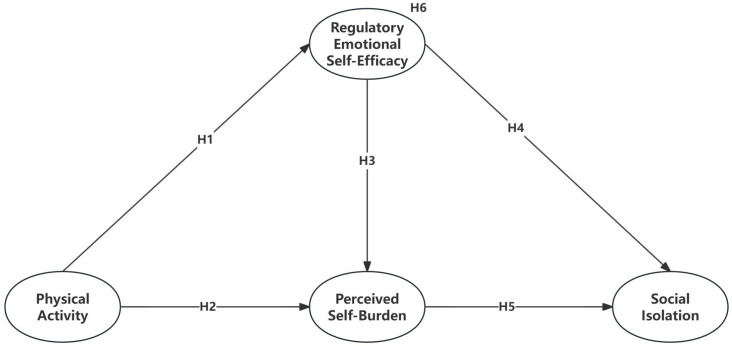
The hypothesized model.

All hypotheses are presented in Figure 1.

## 3. Methods

### 3.1. Procedure

This study was conducted from November to December 2024, targeting elderly individuals aged 60–75 in Changsha. A questionnaire survey was carried out using a combination of snowball and purposive sampling. According to the 2023 Hunan Province Aging Development Statistical Bulletin, the elderly population (aged 60 and above) in Hunan reached 14.6 million by the end of 2023.

This study covered six urban districts in Changsha. Offline survey locations were selected based on the following criteria: (1) the concentration of elderly residents, (2) the availability of community sports facilities, and (3) the proportion of elderly individuals living alone or receiving caregiver assistance. The online survey was conducted via digital communication platforms (WeChat and phone calls) using snowball sampling to expand the sample coverage.

This study adhered to the principle of voluntary participation, ensuring informed consent from all respondents. Personal information was anonymized. As a token of appreciation, each elderly participant received a pack of tissues upon completing the questionnaire. The research team rigorously screened 248 collected questionnaires, excluding 11 invalid responses (e.g., incomplete answers, logical inconsistencies, or duplicate responses), resulting in a final valid response rate of 95.5%.

Table 1 presents the demographic details of the study’s participants, consisting of 237 individuals aged 60 to 75 years. This demographic distribution aligns with the WHO’s categorization, indicating that most participants belong to the younger segment of the elderly population. The sample comprises approximately 47.7% male and 52.3% female participants. Additionally, 74.7% of the elderly participants live with family, while 25.3% live alone. Regarding self-rated health status, 63.7% consider themselves to be in good health. Moreover, about 93.2% of the participants have medical insurance.

### 3.2. Measures

The questionnaire consists of five sections. The first section collects demographic information, including respondents’ age, gender, housing status, self-rated health status, and medical insurance coverage. The second section adopts the scale from Liang [53], comprising three items, such as ‘What is the intensity of physical activity that you usually participate in during the past month?’ This scale demonstrates good internal consistency, with a reliability coefficient of 0.82 [53]. The third section utilizes the scale from Caprara, Di Giunta, Eisenberg, Gerbino, Pastorelli and Tramontano [23], consisting of eight items, including ‘Keep from getting dejected when you are lonely’. While the original scale did not report a Cronbach’s α value, a study by Gore Jr [54] reported a reliability coefficient of 0.88, confirming strong reliability and validity through confirmatory factor analysis. The fourth section employs the scale from Simmons [55], which includes nine items, such as ‘Worry that the health of the caregiver could suffer’. This scale also exhibits strong internal consistency, with a reliability coefficient of 0.938 [55]. Finally, the fifth section adopts the scale from Nicholson Jr et al. [56], containing six items, including ‘See face-to-face at least once a month’. This scale has a reliability coefficient of 0.77, indicating good internal consistency [56]. All four scales are measured using a five-point Likert scale, with response options ranging from 1 (strongly disagree) to 5 (strongly agree).

### 3.3. Data Analysis

After assessing and confirming the quality of the data, responses from 237 participants were used in the final analysis. SPSS 25 software was employed to present demographic data and to clean and prepare the dataset for assumption testing. The proposed research framework was evaluated using structural equation modeling (SEM), with SmartPLS 4.1.0 software specifically applied. The partial least squares (PLS) method was chosen for the constructs and indicators. SEM was then used to examine the model’s validity through convergent and discriminant analyses, calculating the average variance extracted for each construct based on factor loadings [57]. This approach serves as a multivariate analysis technique to explore the complex relationships between variables in the conceptual model [58].

## 4. Results

### 4.1. Information on Physical Activities for the Elderly

Table 2 presents the physical activity data of study participants, including activity intensity, duration, and frequency. Among them, 64.56% reported engaging in light activities such as walking or radio gymnastics, while 16.03% participated in light-to-moderate activities like jogging or Tai Chi.

Regarding activity duration, 12.66% exercised for less than 10 min, 33.33% for 21–30 min, 18.99% for 31–59 min, and 18.14% for over 60 min. In terms of frequency, 11.39% exercised less than once per month, 27.43% engaged in activity 2–3 times per month, 32.49% exercised 1–2 times per week, and 10.97% participated in daily physical activity.

### 4.2. Assessment of the Measurement Model Reliability and Validity

The reliability and validity assessment of latent variables incorporated confirmatory factor analysis (CFA) through SmartPLS 4.1.0. All variables demonstrated Cronbach’s α values surpassing 0.8 (refer to Table 2), affirming robust internal consistency within the model structure as guided by Fornell and Larcker [59]. Additionally, the average variance extraction (AVE) for each variable exceeded 0.6 (as noted in Table 3), surpassing the minimal acceptable threshold of 0.5. Furthermore, the composite reliability (CR) of each latent variable surpassed 0.8, underscoring the model’s robust convergent validity. The resilience of convergent validity across proposed models was well-established. Factor loadings from principal component factor analysis ranged from 0.844 to 0.898 (refer to Table 3), reinforcing the measurement model’s robust construct validity.

In addition, evidence of the discriminant validity for all factors was provided through discriminant verification. Discriminant validity requires statistical tests for components that are not significantly related to other factors when calculating measurement correspondences. It can be assessed by comparing the square root of the AVE with the correlation between factors. The square root of the AVE should be larger than the correlation values. In the discriminant validity analysis, the square roots of all AVEs were higher than the correlation coefficients (see Table 4), indicating a good evaluation.

### 4.3. Hypothesis Testing Results

This study assessed the model fit using the standardized root mean square residual (SRMR), which compares the observed covariance with the hypothesized matrix. SRMR values of 0.08 or lower are generally considered acceptable. The estimated SRMR value in this study was 0.066, indicating a good model fit.

**Figure 2:**
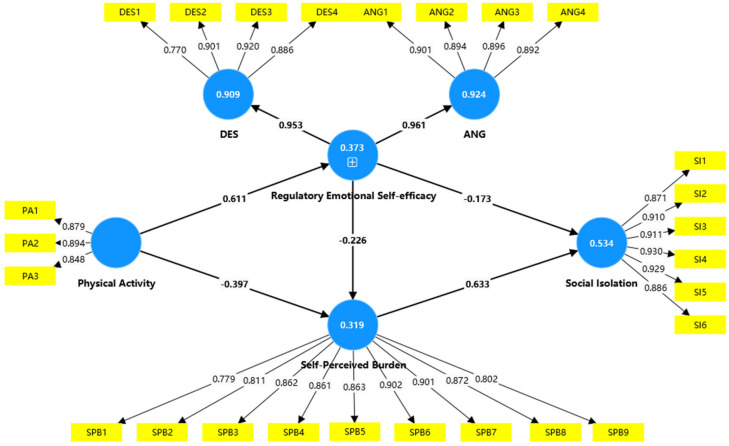
Structural path model.

The structural path model in Table 5 and Figure 2 shows that the relationship between physical activity and regulatory emotional self-efficacy was statistically significant (β = 0.611 t = 15.199, *p* < 0.001), supporting H1; and that there was a significant negative correlation between physical activity and self-perceived burden (β = −0.397, t = 5.013, *p* < 0.001), supporting H2; and that there was a negative correlation between regulatory emotional self-efficacy and self-perceived burden (β = −0.226, t = 2.159, *p* < 0.05), supporting H3; and that there was a negative correlation between regulatory emotional self-efficacy and social isolation (β = −0.173, t = −0.179, *p* < 0.05), supporting H4; and the relationship between self-perceived burden and social isolation was statistically significant (β = 0.633, t = 10.711, *p* < 0.001), supporting H5.

The model’s explanatory power was evaluated by assessing the goodness of fit, determined through the strength of each structural path indicated by the R^2^ value for the dependent variable [60]. According to Falk and Miller [61], an R^2^ value equal to or greater than 0.1 signifies a good fit. As shown in Table 6, all R^2^ values exceeded 0.1, confirming the model’s predictive capability. Additionally, Q^2^ was used to ascertain the predictive relevance of the endogenous constructs; a Q^2^ value above zero indicates predictive relevance. The results in Table 6 demonstrate significant predictive power for the constructs.

### 4.4. Mediation Analysis

The researchers hypothesized that physical activity influences social isolation through two mediators: regulatory emotional self-efficacy and self-perceived burden. This study tested the mediation effects using bootstrapping methods, effect sizes can be assessed through parameters like Cohen’s f^2^, which measure the magnitude of relationships between independent and dependent variables, and a large effect size (f^2^ = 0.35) signifies a strong relationship [62]. Table 7 shows the standardized results of the 5000-bootstrap samples with 95% confidence intervals: no zero values were found within the 95% confidence interval. Moreover, regulatory emotional self-efficacy and self-perceived burden significantly negatively influenced the relationship between physical activity and social isolation (standard indirect effect = −0.445, *p* < 0.001), supporting H6.

## 5. Discussion

### 5.1. Theoretical Contributions

This study contributes to the theoretical understanding of mitigating social isolation among older adults. While existing research primarily addresses social factors influencing social isolation among older adults [63,64], studies on individual-level factors are limited. Therefore, this study focuses on individual-level factors of social isolation among the elderly, highlighting the role of physical activity in alleviating social loneliness. By emphasizing the impact of individual engagement in physical activity, this research contributes to the theoretical development of social isolation studies. The researchers emphasize two primary indicators for assessing social isolation among older adults: psychological well-being and social support, with psychological well-being deemed more crucial than social support [65]. Specifically, this study investigates the impact of physical activity on social isolation among older adults, alongside examining how regulatory emotional self-efficacy and self-perceived burden contribute to social isolation. The findings indicate bidirectional relationships among physical activity, regulatory emotional self-efficacy, self-perceived burden, and social isolation. This study concludes that physical activity enhances regulatory emotional self-efficacy among older adults, mitigates self-perceived burden, consequently reducing the likelihood of social isolation.

Firstly, this study confirms a significant positive correlation between physical activity and regulatory emotional self-efficacy, along with a significant negative correlation with self-perceived burden. This implies that physical activity enhances regulatory emotional self-efficacy and mitigates self-perceived burden, aligning with findings by McAuley et al. [66], emphasizing physical activity’s role in bolstering mental health. Therefore, this study advocates increased sports or exercise participation to foster both physical and mental well-being.

Secondly, regulatory emotional self-efficacy exhibits a significant negative correlation with social isolation, whereas self-perceived burden correlates positively with social isolation. These individual characteristics play pivotal roles in reducing social isolation among older adults through physical activity. While prior research underscores physical activity’s role in diminishing social isolation and fostering social connections [50], this study introduces a novel perspective, suggesting physical activity influences social isolation through regulatory emotional self-efficacy and self-perceived burden.

Finally, regulatory emotional self-efficacy and self-perceived burden mediate the relationship between physical activity and social isolation significantly. By employing regulatory emotional self-efficacy and self-perceived burden as mediators, this study offers a novel hypothetical pathway for understanding the association between physical activity and social isolation. Thus, from an individual characteristic standpoint, this study delves into and elucidates factors influencing social isolation among older adults.

### 5.2. Practical Implications

Given the myriad challenges older adults encounter in daily life, resulting in social isolation, this study recommends leveraging physical activity as a means for them to navigate and mitigate these issues. Previous studies have primarily focused on promoting healthy aging either from an individual perspective or through government policies [67,68]. However, recent studies suggest that a multi-level, collaborative approach may be more effective in addressing the complex factors contributing to social isolation among older adults [69]. Based on this insight, our study proposes a framework involving the government, society, families, and individuals to collectively alleviate social isolation and improve overall well-being.

From a governmental perspective, prior studies have underscored the importance of policy-driven interventions, such as subsidizing fitness programs or integrating physical activity into public health initiatives [10,22,34]. Our findings align with these perspectives but further emphasize the role of regulatory emotional self-efficacy and perceived burden in influencing participation. Thus, in addition to enhancing access to sports facilities, governments should integrate mental health considerations into elderly fitness policies. Collaborating with professional sports organizations to develop tailored, age-friendly exercise programs could further ensure that interventions are both effective and inclusive.

At the social level, the existing literature suggests that community-based physical activities help foster social engagement and reduce loneliness among older adults [19]. However, most studies focus on formal exercise groups (e.g., senior fitness clubs), while our findings indicate that informal physical activities (e.g., Tai Chi, dancing, and light stretching) can also contribute significantly to psychological well-being. Thus, community organizations should expand the range of available activities to accommodate varying physical abilities and interests. Additionally, previous studies have pointed out that social support networks are critical for sustained participation [48], and our study suggests that cross-sector collaboration between community organizations, healthcare providers, and local governments could enhance service delivery and accessibility.

At the family level, relatives should provide maximum encouragement and support for older adults to participate in sports activities, offering necessary support and assistance. Attention should also be paid to the emotional state of older adults, fostering their ability to manage their emotions to reduce issues like anxiety and depression. Additionally, accompanying older adults in various social activities can help expand their social circles and reduce the risk of social isolation.

Lastly, for individuals, it is important to establish a correct health concept, choose sports activities that suit personal conditions and interests, and actively participate to improve physical and mental health. Learning to adjust one’s attitude, maintaining an optimistic and positive approach to life, and enhancing self-emotional management skills are crucial. Importantly, strengthening communication and interaction with family, friends, and society can reduce self-perceived burden and avoid social isolation.

To summarize, our study contributes to the existing body of knowledge by demonstrating that a multi-level, collaborative approach—involving government, society, families, and individuals—is essential to effectively leveraging physical activity as a tool for mitigating social isolation. While previous research has predominantly examined these factors in isolation, our study integrates them into a holistic framework. Future research should continue exploring how these elements interact to optimize intervention strategies for elderly well-being.

### 5.3. Limitations

Firstly, the use of snowball and purposive sampling methods deviates from random sampling, potentially compromising the representativeness of the reported data. Future studies should consider employing probability sampling techniques to enhance generalizability. Secondly, this study does not incorporate additional mediating factors or examine variations across different geographical locations and age groups, which may limit the comprehensiveness of the findings. Future research should explore these aspects to provide a more nuanced understanding of the associations examined. Thirdly, this study only considers two mediating variables—regulatory emotional self-efficacy and perceived burden of self—to investigate the mechanism underlying the impact of physical activity on social isolation. Future research should expand the theoretical framework by incorporating psychosocial and environmental variables (e.g., community engagement, accessibility of sports facilities, and digital interventions for physical activity) to provide a more holistic understanding of the underlying mechanisms. Fourthly, the cross-sectional nature of this study limits the depth and breadth of the data collected. Future investigations should strive to employ longitudinal methods to ensure the thoroughness and scientific integrity of the research findings. For instance, intervention-based studies that implement structured physical activity programs and measure their impact on social isolation and emotional well-being over extended periods could offer more robust insights into the effectiveness of physical activity as an intervention strategy.

## 6. Conclusions

This study addresses the outlined research objectives by highlighting the role of regulatory emotional self-efficacy and perceived burden of self in mitigating social isolation among older adults during physical activities. The findings indicate a significant effectiveness of physical activity in reducing social isolation in this demographic. Notably, physical activity not only directly combats social isolation but also influences its severity through the mediation of regulatory emotional self-efficacy and perceived burden of self.

Therefore, this study recommends that older adults engage in at least two hours of moderate-intensity physical activity per week, based on their individual circumstances. Activities can range from simple recreational exercises, such as walking and jogging, to more intense activities, such as competitive running and swimming. These activities can enhance regulatory emotional self-efficacy, alleviate perceived burden, effectively reduce social isolation, and ultimately improve the overall quality of life for older adults.

## Figures and Tables

**Table 1:** Participant demographics (N = 237).

Title	Profiles	Survey (%)
Gender	Male	113 (47.7%)
	Female	124 (52.3%)
Age	<65	149 (62.9%)
	65–70	80 (33.8%)
	>70	8 (3.3%)
Housing situation	Living alone	60 (25.3%)
	Living with family	177 (74.7%)
Self-assessed health	Well	4 (1.7%)
	Fair	151 (63.7%)
	Poor	82 (34.6%)
Medical insurance	Yes	221 (93.2%)
	No	16 (6.8%)

**Table 2:** Physical activity.

Title	Profiles	Survey (%)
Physical Activity Intensity	Light physical activity (e.g., walking, radio calisthenics)	16.03%
	Light to moderate physical activity (e.g., jogging, Tai Chi)	64.56%
	Moderate to high-intensity physical activity (e.g., cycling, table tennis)	7.59%
	High-intensity physical activity with a short duration (e.g., badminton, tennis)	8.86%
	High-intensity physical activity with a long duration (e.g., swimming, running)	2.95%
Activity Duration	Less than 10 min	12.66%
	11–20 min	16.88%
	21–30 min	33.33%
	31–59 min	18.99%
	60 min or more	18.14%
Activity Frequency	Less than once a month	11.39%
	2–3 times a month	27.43%
	1–2 times a week	32.49%
	3–5 times a week	17.72%
	Daily	10.97%

**Table 3:** Reliability and validity test.

Items	Loading	Cα	CR	AVE
*Physical Activity (PA)*		0.846	0.907	0.764
PA1	0.879	0.797		
PA2	0.894	0.721		
PA3	0.848	0.819		
*Regulatory Emotional Self-Efficacy (RES)*		0.943	0.953	0.716
Perceived Self-Efficacy in Managing Despondency/Distress (DES)		0.893	0.926	0.759
DES1	0.77	0.944		
DES2	0.901	0.934		
DES3	0.92	0.931		
DES4	0.886	0.934		
Perceived Self-Efficacy in Managing Anger/Irritation (ANG)		0.918	0.942	0.803
ANG1	0.901	0.932		
ANG2	0.894	0.934		
ANG3	0.896	0.934		
ANG4	0.892	0.934		
*Self-Perceived Burden (SPB)*		0.952	0.959	0.725
SPB1	0.779	0.950		
SPB2	0.811	0.948		
SPB3	0.862	0.946		
SPB4	0.861	0.946		
SPB5	0.863	0.946		
SPB6	0.902	0.943		
SPB7	0.901	0.944		
SPB8	0.872	0.946		
SPB9	0.802	0.950		
*Social Isolation (SI)*		0.956	0.965	0.822
SI1	0.871	0.953		
SI2	0.910	0.947		
SI3	0.911	0.947		
SI4	0.930	0.944		
SI5	0.929	0.944		
SI6	0.886	0.951		

Note: Cα = Cronbach’s alpha; AVE = average variance extracted; CR = composite reliability.

**Table 4:** Display discriminant validity analysis.

	RES	PA	SPB	SI
RES	0.846			
PA	0.611	0.874		
SPB	−0.469	−0.535	0.851	
SI	−0.470	−0.532	0.714	0.906

Note: PA = physical activity; RES = regulatory emotional self-efficacy; SPB = self-perceived burden; SI = social isolation.

**Table 5:** Path coefficients.

No.	Path	β	SEs	T Statistics	*p* Values	LLIC	ULIC
H1	PA → RES	0.611	0.612	15.199	0.000	0.529	0.687
H2	PA → SPB	−0.397	−0.395	5.013	0.000	−0.543	−0.237
H3	RES → SPB	−0.226	−0.231	2.159	0.031	−0.433	−0.026
H4	RES → SI	−0.173	−0.179	2.544	0.011	−0.318	−0.055
H5	SPB → SI	0.633	0.629	10.711	0.000	0.503	0.731

Note: PA = physical activity; RES = regulatory emotional self-efficacy; SPB = self-perceived burden; SI = social isolation.

**Table 6:** Outcomes of R^2^ and Q^2^ values.

	R^2^	Adjusted R^2^	Q^2^
RES	0.373	0.370	0.264
SPB	0.319	0.313	0.226
SI	0.534	0.530	0.433

RES = regulatory emotional self-efficacy; SPB = self-perceived burden; SI = social isolation.

**Table 7:** Mediation analysis.

No	Path	Effect	Boot SE	T Statistics	*p* Values	Boot LLIC	Boot ULIC
H6	Total indirect effect of PA → SI	−0.445	−0.447	8.557	0.000	−0.547	−0.344
	Total Effect of PA → SI	−0.445	−0.447	8.557	0.000	−0.547	−0.344

Note: PA = physical activity; SI = social isolation.

## Data Availability

The data used to support the findings of this study are available from the corresponding author upon request.
